# The anxiety of nursing professionals during the COVID-19 pandemic period in a brazilian regional university hospital

**DOI:** 10.1192/j.eurpsy.2021.809

**Published:** 2021-08-13

**Authors:** L. Floriano, E. Dal’Bosco, R. Plantes, G. Arcaro, M. Ribas, E. Krum

**Affiliations:** 1 Nursing And Public Health, State University of Ponta Grossa, Ponta Grossa, Brazil; 2 Pharmacy, State University of Ponta Grossa, Ponta Grossa, Brazil

**Keywords:** mental health, COVID-19, Health promotion

## Abstract

**Introduction:**

Nursing work requires technical, scientific competence, knowledge, skill and emotional control over practice, considering that care presents risk situations, physical and emotional stress, responsibilities with people’s lives, coping with fears and suffering. All this situation in which the professional is exposed can lead to the occurrence of psychological wear, high stress and anxiety, this is conceptualized as a vague and unpleasant feeling of fear, apprehension, with characteristics of tension or discomfort derived from anticipating danger, something unknown or strange.

**Objectives:**

To identify the prevalence and factors associated with anxiety among nursing professionals who work coping with COVID-19 in a Brazilian regional university hospital.

**Methods:**

Cross-sectional observational study, with sociodemographic questionnaire and anxiety measurement scale (HAD), with 88 nursing professionals. The data were analyzed using absolute and relative frequency, using the software StatisticalPackage for the Social Sciences.

**Results:**

There was a prevalence of anxiety (48.9%), with the majority of the sample consisting of women, over 40 years old, married or in a stable relationship, white, with higher education or postgraduate education, with income above R $ 3,000.00, tendered, with a work regime of 40 hours per week and time in the hospital from 1 to 5 years.

**Conclusions:**

The impact should be considered on Nursing Mental Health caused by COVID-19 and intervene with coping strategies to minimize anxiety.

